# Correction: Xu et al. Detection of Alpha- and Betacoronaviruses in Small Mammals in Western Yunnan Province, China. *Viruses* 2023, *15*, 1965

**DOI:** 10.3390/v16101617

**Published:** 2024-10-16

**Authors:** Fen-Hui Xu, Pei-Yu Han, Jia-Wei Tian, Li-Dong Zong, Hong-Min Yin, Jun-Ying Zhao, Ze Yang, Wei Kong, Xing-Yi Ge, Yun-Zhi Zhang

**Affiliations:** 1 School of Public Health, Institute of Preventive Medicine, Dali University, Dali 671000, China; xufenhui1569@163.com (F.-H.X.); hanpeiyu1511@gmail.com (P.-Y.H.); tjwfatorange@outlook.com (J.-W.T.); zld2019106326@163.com (L.-D.Z.); 13099916216@163.com (H.-M.Y.); zhaojunying0714@163.com (J.-Y.Z.); yangzeyz@163.com (Z.Y.); kongwei4357@163.com (W.K.); 2 Key Laboratory of Pathogen Resistant Plant Resources Screening Research in Western Yunnan, Dali 671000, China; 3 Key Laboratory of Cross-Border Prevention and Control and Quarantine of Zoonotic Diseases in Yunnan, Dali 671000, China; 4 College of Biology & Hunan Provincial Key Laboratory of Medical Virology, Hunan University, Changsha 410012, China; xyge@hnu.edu.cn

## Error in Table

In the original publication [1], there was a mistake in Table 2. The species name “Short-tailed shrew (*Blarina brevicauda*)” should be corrected to “Chinese mole shrew (*Anourosorex squamipes*)”. The corrected “Table 2” appears below. The authors state that the scientific conclusions are unaffected. This correction was approved by the Academic Editor. The original publication has also been updated.

## Figures and Tables

**Table 2:** The situation of CoV infection in small mammals in Dali and Nujiang prefectures, Yunnan Province.

Order	Species	Locations	Composition, %	Prevalence, %		
				qRT-PCR	RT-PCR	
					α-CoV	β-CoV
*Rodentia*	Chevrieri’s field mouse (*Apodemus chevrieri*)	Dali, Nujiang	22.51 (113/502)	9.73 (11/113)	0 (0/113)	3.54 (4/113)
	Lancangjiang field mouse (*Apodemus ilex*)	Dali, Nujiang	17.93 (90/502)	21.11 (19/90)	0 (0/90)	6.67 (6/90)
	Kachin red-backed vole (*Eothenomys cachinus*)	Dali, Nujiang	16.14 (81/502)	11.11 (9/81)	2.22 (2/90)	2.22 (2/90)
	Large oriental vole (*Eothenomys miletus*)	Dali, Nujiang	7.77 (39/502)	12.82 (5/39)	0 (0/39)	0 (0/39)
	Asian house rat (*Rattus tanezumi*)	Dali, Nujiang	14.94 (75/502)	25.33 (19/75)	0 (0/75)	0 (0/75)
	Norway rat (*Rattus norvegicus*)	Dali	5.18 (26/502)	46.15 (12/26)	3.85 (1/26)	3.85 (1/26)
	Grey bellied mouse (*Niviventer eha*)	Nujiang	0.6 (3/502)	0 (0/3)	0 (0/3)	0 (0/3)
	Ryukyu mouse (*Mus caroli*)	Dali	0.6 (3/502)	0 (0/3)	0 (0/3)	0 (0/3)
	House mouse (*Mus musculus*)	Dali	0.40 (2/502)	0 (0/2)	0 (0/2)	0 (0/2)
	Chestnut white-bellied rat (*Niviventer fulvescens*)	Dali, Nujiang	0.40 (2/502)	0 (0/2)	0 (0/2)	0 (0/2)
	White-footed Indochinese rat (*Rattus nitidus*)	Dali	0.80 (4/502)	75 (3/4)	75 (3/4)	0 (0/4)
	Swinhoe’s striped squirrel *(Tamiops swinhoei*)	Dali	0.20 (1/502)	0 (0/1)	0 (0/1)	0 (0/1)
*Insectivora*	Chinese mole shrew (*Anourosorex squamipes*)	Nujiang	4.18 (21/502)	4.67 (1/21)	0 (0/21)	0 (0/21)
	Long-tailed red-toothed shrew (*Episoriculus leucops*)	Nujiang	3.39 (17/502)	5.88 (1/17)	5.88 (1/17)	0 (0/17)
	Asian gray shrew *(Crocidura attenuata*)	Nujiang	2.19 (11/502)	27.27 (3/11)	0 (0/11)	0 (0/11)
	House musk shrew (*Suncus murinus*)	Dali	0.60 (3/502)	66.67 (2/3)	0 (0/3)	0 (0/3)
*Lagomorpha*	Tibet pika (*Ochotona thibetana*)	Nujiang	1.79 (9/502)	11.11 (1/9)	0 (0/9)	0 (0/9)
*Scandentia*	Tree shrew (*Tupaia belangeri*)	Dali	0.40 (2/502)	50 (1/2)	0 (0/2)	0 (0/2)
	Total		100 (502/502)	17.33 (87/502)	1.39 (7/502)	2.59 (13/502)
