# Supplementary material for: Detection of Alpha- and Betacoronaviruses in Small Mammals in Western Yunnan Province, China
Source: Viruses. 2023 Sep 20;15(9):1965. doi: 10.3390/v15091965 (PMC10535241; doi:10.3390/v15091965)
Supplement: Supplementary file 1 [file viruses-15-01965-s001.zip › Table S1.pdf]

Table S1. Partial RdRp nucleotide and amino acid sequence identity alignment of β-CoV *.

	CoVD L55	CoVD L75	CoVD L140	CoVD L161	CoVD L172	CoV NJ16	CoV NJ21	CoV NJ33	CoV NJ53	CoV NJ55	CoV NJ56	CoV NJ99	CoVN J142	Lijian g-41	Ruili-8 74	RtAp-CoV/ SAX2015	Lijian g-53	BOV-36/IN D/2015	DcCoV-H KU23
CoVDL55		99.40	99.35	99.57	98.89	99.28	100.00	97.14	100.00	100.00	94.66	91.43	90.14	97.54	96.86	53.08	99.80	49.36	49.36
CoVDL75	98.55		99.14	98.92	98.52	99.28	100.00	97.14	100.00	100.00	94.66	91.43	90.14	97.34	96.65	53.08	99.60	49.36	49.36
CoVDL140	96.56	96.77		98.92	98.89	99.28	100.00	97.14	100.00	100.00	94.66	91.43	90.14	98.92	99.78	55.87	99.57	51.95	51.95
CoVDL161	99.00	98.92	96.56		98.52	98.55	100.00	96.43	99.25	100.00	93.89	90.71	89.44	98.71	99.14	55.47	99.35	51.54	51.54
CoVDL172	98.65	98.15	95.33	97.91		97.10	98.72	94.47	97.74	98.72	92.37	90.14	89.29	98.52	98.89	74.04	98.89	68.56	68.56
CoVNJ16	97.59	96.87	96.63	96.39	95.90		76.83	96.38	99.25	76.83	95.84	90.58	90.58	98.55	99.28	99.28	99.28	91.30	91.30
CoVNJ21	98.72	100.00	98.29	100.00	98.46	96.92		78.66	78.66	100.00	75.00	65.85	62.79	100.00	100.00	78.66	100.00	65.85	65.85
CoVNJ33	97.14	96.43	95.24	95.95	95.19	96.54	98.20		96.99	78.66	93.51	88.57	88.57	96.43	97.14	96.63	97.14	89.29	89.29
CoVNJ53	98.75	97.99	96.99	97.74	97.24	97.74	98.20	98.75		78.66	96.62	90.98	90.98	99.25	100.00	100.00	100.00	91.73	91.73
CoVNJ55	99.23	98.46	98.46	98.46	98.46	96.92	98.46	99.74	99.74		75.00	65.85	62.79	100.00	100.00	78.66	100.00	65.85	65.85
CoVNJ56	96.63	95.87	95.36	95.62	95.11	95.25	98.46	95.68	96.97	98.46		88.05	88.55	93.89	94.66	96.62	94.66	88.83	88.83
CoVNJ99	84.29	83.57	84.76	83.10	82.45	83.99	80.41	84.05	84.46	81.19	83.75		100.00	90.71	91.43	90.87	91.43	99.29	99.29
CoVNJ142	83.42	82.73	84.57	82.51	82.49	83.86	66.60	83.81	84.46	80.77	82.49	98.57		89.44	90.14	90.43	90.14	98.82	98.82
Lijiang-41	98.35	98.35	96.63	98.21	97.17	96.87	99.15	96.43	98.25	98.46	96.12	83.57	82.96		99.14	99.28	97.75	91.43	91.43
Ruili-874	94.19	94.40	99.57	96.85	95.69	96.39	98.72	95.48	97.24	99.23	95.62	85.00	84.80	94.26		100.00	99.78	92.14	92.14
RtAp-CoV/SAX2015	51.86	51.47	54.34	54.20	72.85	98.07	77.73	96.15	97.99	98.46	96.29	84.62	83.65	97.35	97.35		100.00	92.03	92.03
Lijiang-53	95.31	95.79	97.13	98.28	97.17	97.11	98.72	96.19	97.74	97.69	95.62	83.57	82.73	95.51	97.42	97.11		92.14	92.14
BOV-36/IND/2015	45.13	44.75	48.32	47.11	62.71	84.47	63.41	83.57	84.71	81.19	84.01	98.33	97.34	84.05	85.95	85.44	83.57		100.00
DcCoV-HKU23	46.75	46.38	49.85	48.66	64.53	84.58	68.08	83.81	84.71	81.54	84.88	98.10	97.86	84.67	86.50	85.30	84.44	99.52	

*The upper right is the amino acid sequence alignment result; The bottom left is the nucleotide sequence alignment result.
