# Supplementary material for: Detection of Alpha- and Betacoronaviruses in Small Mammals in Western Yunnan Province, China
Source: Viruses. 2023 Sep 20;15(9):1965. doi: 10.3390/v15091965 (PMC10535241; doi:10.3390/v15091965)
Supplement: Supplementary file 1 [file viruses-15-01965-s001.zip › Table S2.pdf]

Table S2. Partial RdRp nucleotide and amino acid sequence identity alignment of α-CoV *.

	CoVDL82	CoVNJ3	CoVNJ52	CoVNJ135	CoVNJ195	CoVNJ196	CoVNJ207	RtRl-CoV/FJ2015	RtClan-CoV/GZ2015
CoVDL82		94.44	95.01	98.76	99.31	97.22	96.93	100.00	97.14
CoVNJ3	94.00		97.26	93.83	93.75	93.06	91.98	94.10	96.93
CoVNJ52	95.73	99.25		95.06	94.26	93.52	95.21	95.97	98.99
CoVNJ135	99.18	92.56	93.39		97.53	96.30	97.53	98.76	96.30
CoVNJ195	98.61	93.14	95.23	96.96		97.92	97.64	98.35	95.52
CoVNJ196	97.69	93.14	94.97	96.20	98.85		96.93	97.64	94.81
CoVNJ207	98.25	94.11	95.23	96.32	99.44	99.68		97.86	95.00
RtRl-CoV/FJ2015	98.77	93.23	95.48	98.56	98.77	98.08	98.97		97.14
RtClan-CoV/GZ2015	95.77	96.23	98.74	94.68	94.85	94.15	94.91	96.08	

*The upper right is the amino acid sequence alignment result; The bottom left is the nucleotide sequence alignment result.
