# Supplementary material for: Detection of Alpha- and Betacoronaviruses in Small Mammals in Western Yunnan Province, China
Source: Viruses. 2023 Sep 20;15(9):1965. doi: 10.3390/v15091965 (PMC10535241; doi:10.3390/v15091965)
Supplement: Supplementary file 1 [file viruses-15-01965-s001.zip › Table S3.pdf]

Table S3. Intra group repeatability test results.

	Number of copies/(copies/μL)	Ct values			Mean Ct	SD	CV,%
		1	2	3			
α-CoV	$1.00{\times}10^9$	14.70	14.75	14.63	14.69	0.06	0.41
	$1.00{\times}10^8$	17.90	17.78	17.83	17.84	0.06	0.33
	$1.00{\times}10^7$	20.96	20.79	20.98	20.91	0.10	0.49
	$1.00{\times}10^6$	23.70	23.67	23.59	23.65	0.06	0.26
	$1.00{\times}10^5$	27.44	27.49	27.21	27.38	0.30	1.09
	$1.00{\times}10^4$	31.15	30.70	30.72	31.03	0.24	0.78
β-CoV	$1.00{\times}10^9$	13.15	13.31	13.14	13.2	0.1	0.74
	$1.00{\times}10^8$	16.99	16.83	16.97	16.93	0.09	0.52
	$1.00{\times}10^7$	20.53	20.05	20.09	20.22	0.27	1.33
	$1.00{\times}10^6$	23.03	23.75	23.15	23.31	0.39	1.66
	$1.00{\times}10^5$	27.11	27.14	27.60	27.28	0.27	1.00
	$1.00{\times}10^4$	30.59	30.53	30.63	30.58	0.05	0.16
