# Supplementary material for: Detection of Alpha- and Betacoronaviruses in Small Mammals in Western Yunnan Province, China
Source: Viruses. 2023 Sep 20;15(9):1965. doi: 10.3390/v15091965 (PMC10535241; doi:10.3390/v15091965)
Supplement: Supplementary file 1 [file viruses-15-01965-s001.zip › Table S4.pdf]

Table S4. Inter group repeatability test results.

	Number of copies/(copies/μL)	Ct values 1	2	3	Mean Ct	SD	CV,%
α-CoV	1.00×10^9	14.58	14.33	14.35	14.42	0.14	0.96
	1.00×10^8	15.33	15.38	15.57	15.42	0.13	0.81
	1.00×10^7	19.30	19.01	19.19	19.17	0.15	0.78
	1.00×10^6	22.38	22.37	22.36	22.37	0.01	0.03
	1.00×10^5	25.93	25.90	25.89	25.83	0.02	0.07
	1.00×10^4	34.45	34.15	34.39	34.33	0.16	0.46
β-CoV	1.00×10^9	14.98	14.91	14.91	14.93	0.04	0.28
	1.00×10^8	16.62	16.74	16.66	16.67	0.06	0.38
	1.00×10^7	18.92	18.74	18.74	18.8	0.1	0.55
	1.00×10^6	23.46	23.54	23.56	23.52	0.05	0.22
	1.00×10^5	27.22	26.57	26.30	26.7	0.47	1.77
	1.00×10^4	32.26	31.50	31.87	31.88	0.38	1.19
