# Supplementary material for: Detection of Alpha- and Betacoronaviruses in Small Mammals in Western Yunnan Province, China
Source: Viruses. 2023 Sep 20;15(9):1965. doi: 10.3390/v15091965 (PMC10535241; doi:10.3390/v15091965)
Supplement: Supplementary file 1 [file viruses-15-01965-s001.zip › Table S5.pdf]

Table S5. Basic information on the positive samples and reference strains obtained in this study.

	Sequence	Type	Location	Collection date	Host	Tissue type	Genbank accession number
positive samples obtained in this study	CoVDL55	β-CoV	Heqing County-Dali Prefecture-Yunnan Province-China(arable areas)	7-Jul-21	*Apodemus chevrieri*	rectum	OR223161
	CoVDL75	β-CoV	Heqing County-Dali Prefecture-Yunnan Province-China(arable areas)	31-Jul-21	*Apodemus chevrieri*	rectum	OR223162
	CoVDL82	α-CoV	Dali City-Dali Prefecture-Yunnan Province-China(arable areas)	15-Dec-21	*Rattus norvegicus*	rectum	OR223163
	CoVDL140	β-CoV	Dali City-Dali Prefecture-Yunnan Province-China(arable areas)	12-Jan-22	*Rattus norvegicus*	rectum	OR223164
	CoVDL161	β-CoV	Dali City-Dali Prefecture-Yunnan Province-China(arable areas)	18-Jan-22	*Apodemus chevrieri*	rectum	OR223165
	CoVDL172	β-CoV	Dali City-Dali Prefecture-Yunnan Province-China(arable areas)	19-Jan-22	*Apodemus chevrieri*	rectum	OR223166
	CoVNJ3	α-CoV	Lushui City-Nujiang Prefecture-Yunnan Province-China(wild bush areas)	20-Jul-22	*Eothenomys cachinus*	rectum	OR223167
	CoVNJ16	β-CoV	Gongshan County-Nujiang Prefecture-Yunnan Province-China(wild bush areas)	1-Aug-22	*Apodemus ilex*	rectum	OR223168
	CoVNJ21	β-CoV	Gongshan County-Nujiang Prefecture-Yunnan Province-China(wild bush areas)	1-Aug-22	*Apodemus ilex*	rectum	OR223169
	CoVNJ33	β-CoV	Gongshan County-Nujiang Prefecture-Yunnan Province-China(wild bush areas)	1-Aug-22	*Apodemus ilex*	rectum	OR223170
	CoVNJ52	α-CoV	Lushui City-Nujiang Prefecture-Yunnan Province-China(wild bush areas)	21-Jul-22	*Eothenomys cachinus*	rectum	OR223171
	CoVNJ53	β-CoV	Gongshan County-Nujiang Prefecture-Yunnan Province-China(wild bush areas)	1-Aug-22	*Apodemus ilex*	rectum	OR223172
	CoVNJ55	β-CoV	Gongshan County-Nujiang Prefecture-Yunnan Province-China(wild bush areas)	1-Aug-22	*Apodemus ilex*	rectum	OR223173
	CoVNJ56	β-CoV	Gongshan County-Nujiang Prefecture-Yunnan Province-China(wild bush areas)	1-Aug-22	*Apodemus ilex*	rectum	OR223174
	CoVNJ99	β-CoV	Gongshan County-Nujiang Prefecture-Yunnan Province-China(wild bush areas)	2-Aug-22	*Eothenomys cachinus*	rectum	OR223175
	CoVNJ135	α-CoV	Gongshan County-Nujiang Prefecture-Yunnan Province-China(wild bush areas)	2-Aug-22	*Episoriculus leucops*	rectum	OR223176
	CoVNJ142	β-CoV	Gongshan County-Nujiang Prefecture-Yunnan Province-China(wild bush areas)	2-Aug-22	*Eothenomys cachinus*	rectum	OR223177
	CoVNJ195	α-CoV	Gongshan County-Nujiang Prefecture-Yunnan Province-China(residential areas)	6-Aug-22	*Rattus nitidus*	rectum	OR223178
	CoVNJ196	α-CoV	Gongshan County-Nujiang Prefecture-Yunnan Province-China(residential areas)	6-Aug-22	*Rattus nitidus*	rectum	OR223179
	CoVNJ207	α-CoV	Gongshan County-Nujiang Prefecture-Yunnan Province-China(residential areas)	6-Aug-22	*Rattus nitidus*	rectum	OR223180
reference strains	Lijiang-41	β-CoV	Lijiang City-Yunnan Province-China	2015	*Apodemus latronum*	-	MT820628
	Lijiang-53	β-CoV	Lijiang City-Yunnan Province-China	2014	*Apodemus chevrieri*	-	MT820629
	Ruili-874	β-CoV	Ruili City-Yunnan Province-China	2014	*Rattus tanezumi*	-	MT820631
	RtAp/SAX2015	β-CoV	Shanxi Province-China	Jun-15	*Apodemus peninsulae*	-	KY370064
	BOV-36/IND/2015	β-CoV	India	2015	*bovine*	feces	MH753496
	DcCoV-HKU23	β-CoV	Morocco	4-Apr-16	*Camelus dromedarius*	Nasal swab	MN514976
	RtRl/FJ2015	α-CoV	Fujian Province-China	15-May	*Rattus losea*	pharyngeal and anal swabs	KY370050
	RtClan/GZ2015	α-CoV	Guizhou Province-China	15-Dec	*Eothenomys melanogaster*	pharyngeal and anal swabs	KY370054
